# The South African dysphagia screening tool (SADS): A screening tool for a developing context

**DOI:** 10.4102/sajcd.v63i1.117

**Published:** 2016-02-16

**Authors:** Calli Ostrofsky, Jaishika Seedat

**Affiliations:** 1 Department of Speech and Hearing Therapy, University of Witwatersrand, South Africa

## Abstract

**Background:**

Notwithstanding its value, there are challenges and limitations to implementing a dysphagia screening tool from a developed contexts in a developing context. The need for a reliable and valid screening tool for dysphagia that considers context, systemic rules and resources was identified to prevent further medical compromise, optimise dysphagia prognosis and ultimately hasten patients’ return to home or work.

**Methodology:**

To establish the validity and reliability of the South African dysphagia screening tool (SADS) for acute stroke patients accessing government hospital services. The study was a quantitative, non-experimental, correlational cross-sectional design with a retrospective component. Convenient sampling was used to recruit 18 speech-language therapists and 63 acute stroke patients from three South African government hospitals. The SADS consists of 20 test items and was administered by speech-language therapists. Screening was followed by a diagnostic dysphagia assessment. The administrator of the tool was not involved in completing the diagnostic assessment, to eliminate bias and prevent contamination of results from screener to diagnostic assessment. Sensitivity, validity and efficacy of the screening tool were evaluated against the results of the diagnostic dysphagia assessment. Cohen’s kappa measures determined inter-rater agreement between the results of the SADS and the diagnostic assessment.

**Results and conclusion:**

The SADS was proven to be valid and reliable. Cohen’s kappa indicated a high inter-rater reliability and showed high sensitivity and adequate specificity in detecting dysphagia amongst acute stroke patients who were at risk for dysphagia. The SADS was characterised by concurrent, content and face validity. As a first step in establishing contextual appropriateness, the SADS is a valid and reliable screening tool that is sensitive in identifying stroke patients at risk for dysphagia within government hospitals in South Africa.

## Introduction

There are many challenges surrounding effective identification of patients who present with dysphagia within government hospitals in developing contexts. Some of these challenges include timing of identification of a swallowing difficulty, timing of the assessment and use of non-standardised contextualised protocols for assessment and intervention (Seedat, 2013). The lack of standardisation contributes to variable service delivery from one patient to the next. This is problematic. For decades, speech-language therapists (SLTs) from less developed contexts have relied on internationally developed tools to guide assessment and intervention in dysphagia, as these had a research and evidence-based underpinning (Emanuel, Wendler, Killen & Grady, 2004). Two key factors have necessitated a revision of this almost standard protocol of merely implementing internationally developed tools on the South African population.

Firstly with disease profiles, unemployment rates, dietary intake, accessibility of services and socio-economic status changing dramatically over the last two decades within countries across the world, it is becoming increasingly difficult to compare patient profiles from developed contexts with those from less developed and developing contexts (Mamdani, 2011). Direct application and use of guidelines without any tailoring or modification is becoming increasingly inappropriate and inadequate to meet the needs of the clients from less developed contexts. Linked to this is the status of government health care institutions in developing contexts. In South Africa, financial restrictions and budgets dictate what equipment, if any, hospitals have (Cullinan, 2006). Challenges around availability of equipment, malfunctioning equipment, stolen equipment and servicing of equipment remain significant obstacles with dysphagia service provision in government hospitals. Having necessary objective measures in dysphagia intervention such as videofluoroscopy is considered a luxury, with even basic consumables, such as mouth care kits for oral hygiene, often unavailable.

Secondly, whilst knowledge and awareness around swallowing and swallowing impairments have improved, this has an implication for the workloads of SLTs employed at hospitals. Regardless of its status as a priority for hospital-based SLTs, dysphagia is but one amongst an array of other speech therapy services patients at hospitals require (American Speech-Language and Hearing Association, 2013; Health Professions Council of South Africa, 1988; South African Speech-language and Hearing Association, 2009). Consequently time, human resources and efficiency of services are compromised for the increasing number of patients that need to be seen who present with dysphagia as well as other speech and language communication disorders. This has also been influenced by the increasing prevalence of chronic disorders such as strokes and HIV, cancer (head and neck) and degenerative neurologic conditions as a result of lifestyle changes, eating habits and poor medical follow-up (Blackwell & Littlejohns, 2010; Brainin, Teuschl & Kalra, 2007; Connor *et al*., 2008; Crary *et al*., 2013).

Other reasons limiting direct implementation of internationally developed tools pertain to institutional ‘culture’ amongst health care institutions in South Africa. Multidisciplinary team management is not ideal (Martens, Cameron & Simonsen, 1990; Seedat, 2013). Hence, it remains challenging to rely on other health professionals as part of team management due to poor role clarity and limited knowledge of each other’s role and responsibilities when working with the patient with dysphagia (Blackwell & Littlejohns, 2010). A need for a contextualised, specific, valid and reliable dysphagia screening tool that could address some of the aforementioned barriers was identified.

## Intervening early with dysphagia

Dysphagia, characterised by difficulty with the passage of food from the mouth to the stomach, is defined as a swallowing disorder affecting the oral, pharyngeal or oesophageal phase of swallowing (Falsetti *et al*., 2009). Dysphagia is commonly a symptom of neurological disease such as stroke (Blackwell & Littlejohns, 2010). The association between stroke and dysphagia has been established, with reports ranging from one-third to two-thirds of acute stroke patients presenting with dysphagia (Perry, 2000). Whilst exact statistics on the incidence of dysphagia amongst stroke patients in South Africa are not readily available, one may surmise that given the increasing prevalence of strokes (Connor & Bryer, 2006; Connor *et al*., 2008), there is likely a consequent increase in associated dysphagia.

Early evaluation of dysphagia amongst stroke patients may decrease one’s vulnerability to co-morbidities (Hinchey *et al*., 2005), such as aspiration pneumonia, malnutrition, dehydration, airway obstruction or even death (Perry, 2000). Additional considerations for the patient accessing a government hospital in South Africa are (1) financial implications – patients who are breadwinners of the family need to return to work and increased length of hospitalisation impacts this – and (2) an increased likelihood of the patient acquiring other hospital-acquired infections and co-morbidities.

Early detection facilitates optimal management, minimises occurrence of dysphagia-related complications, as well as other co-morbidities, and can improve the prognosis for dysphagia (Blackwell & Littlejohns, 2010; Heckert, Komaroff, Adler & Barrett, 2009; Marik & Kaplan, 2003; Martino, Pron & Diamant, 2000). Cost efficiency for the hospital department, for the hospital and for the patient are important considerations in any resource-constrained and developing context.

## The need for a contextually relevant and valid dysphagia screening tool

A standardised screening tool facilitates identification of a disorder (Martino *et al*., 2000). Many screening tools for dysphagia with established reliability and validity exist (Martino *et al*., 2009; Trapl *et al*., 2007). Mamdani (2011), however, cautions against readily accepting and implementing tools developed in First World countries to populations in developing Second and Third World countries. As noted above, populations, environments, systems and so on may be very different, necessitating modification of internationally developed tools (Mamdani, 2011).

It is important that a screening tool considers context-specific variables, resources, logistics and systemic rules. The high demand on health care professionals and the government health care system itself increases the likelihood of subtle problems, further medical compromise and delayed identification of dysphagia only when the patient is at a critical stage or when the dysphagia becomes more ‘overt’ to the casual observer. The availability and use of a context-appropriate screening tool can relieve the demand on staff, resources and time through its simplicity, whilst maximising dysphagia detection. As it stands, implications for early, reliable and efficient dysphagia identification and management within this context, given the challenges, are discouraging. Competency, knowledge and commitment of the administrator of the screening tool must be given consideration in the development of a contextually relevant and appropriate tool. Optimal features of a screening tool are presented in Table 1.

**TABLE 1:** Guidelines to adhere to when developing a screening tool.

Guidelines
Reliable
Sensitive
Specific
Valid
Quick to administer
Easy to understand terminology
Easy to administer
Resource conservative
Acceptable to patients
Context appropriateness

*Source*: Cochrane & Holland, 1971, as cited in Perry, 2000; Hinds & Wiles, 1998; Perry & Love, 2001; Heart and Stroke Foundation of Ontario, 2006

### Problem statement

The availability of numerous screening tools, each validated and proven reliable, raised the question of the need to develop yet another screening tool. The dilemma of which existing screening tool to validate on the South African population led to the development of a new screening tool. The new tool incorporated the proven benefits of existing tools whilst heeding their limitations and simultaneously ensuring cultural and linguistic sensitivity for the general South African population. It also considered the realities of acute hospital wards, the staff available, time available and access to resources. However, the question remained: would the newly developed dysphagia screening tool facilitate valid and reliable identification of dysphagia, amongst patients presenting with stroke as their underlying medical pathology?

## Methodology

### Aim

To assess the reliability and validity of a newly developed dysphagia screening tool to identify dysphagia in acute adult patients presenting with stroke.

### Design

A quantitative research methodology using a non-experimental, correlational cross-sectional design was used. A retrospective component was necessary to review patient files after administration of a diagnostic dysphagia assessment. This is discussed below.

### Process and sample

Three government hospitals in South Africa were the research sites. Necessary ethical approval was obtained from the University of Witwatersrand Human Research and Ethics Committee (Medical; protocol no. M120215). There were two cohorts of participants: 18 SLTs and 63 stroke patients. Convenience sampling was used to recruit both cohorts. The participants with dysphagia were recruited by the recruited SLT participants. The patient sample adequately represented patients attending government hospitals in South Africa from varying financial, sociolinguistic and cultural backgrounds. The patient sample received the SADS (dysphagia screening) and following this, within 24 hours, a diagnostic dysphagia assessment. 63 patients were screened and 62 received a diagnostic dysphagia assessment. The SLT sample were clinicians working within the government hospitals from which the patient sample was recruited. The clinician sample was responsible for conducting the SADS and the diagnostic dysphagia assessment. Although they may be regarded as research assistants, they have been described as a participant sample, as they had to adhere to inclusion and exclusion criteria and be recruited by employing convenience sampling. Further, it was necessary for the SLTs to consent to participate in the study.

### Data collection

**FIGURE 1:**
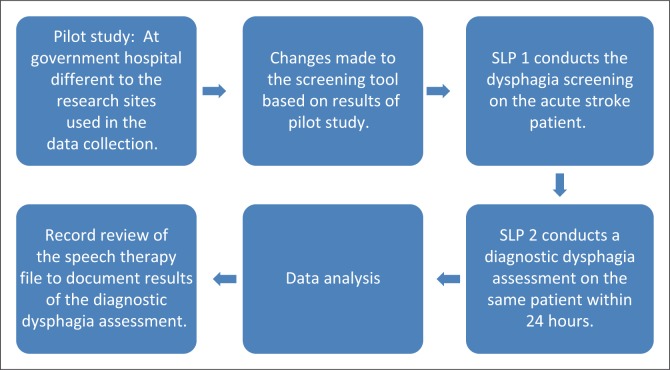
Process followed for data collection

Figure 1 provides an illustration of the procedure that was followed for data collection.

The pilot study evaluated aspects of the design and procedure of the research, as well as the developed research tool in terms of feasibility, usefulness and ease of administration (Clark-Carter, 2010; McBurney & White, 2010).

## The screening tool (refer to Appendix 1)

The South African Dysphagia Screening (SADS) tool consists of four sections and 20 test items. The items within each subsection are described below.

### Section 1

The items in section 1 were aimed at determining the alertness of the stroke participant. A patient’s compromised state of consciousness may have implications on their ability to swallow safely or alert the SLT to possible swallowing difficulty that is the patient is experiencing (Martino *et al*., 2009).

### Section 2

Items in this section provide subjective interpretations of the screening results. Regardless of the answer, items in section 2 do not categorise the patient as a ‘refer’ but need to be considered.

The first item in section 2 evaluates the patient’s position. According to Tanner (2007), swallowing whilst seated in an upright position achieves maximum protection of the airway and reduces the chances of aspiration. Thus, incorrect positioning may compromise airway protection.

The second item in section 2 requires the patient to count. This item serves a two-fold function. It allows the SLT to gain an impression of the patient’s voice, as well as insight into the patient’s receptive language understanding. Assessment of swallowing requires patients to follow instructions (e.g. open your mouth), be alert and have a degree of understanding that will enable an appropriate assessment. Inability to understand simple instructions may have implications on ability to swallow safely or alert the SLT to possible swallowing difficulties that the patient may be experiencing (Martino *et al*., 2009).

Voicing provides information on laryngeal functioning (Cichero & Murdoch, 2006). Careful attention is to be directed to the patient’s voice quality prior to and after each delivery of food or liquid in order to ascertain whether food is entering the larynx, thus potentially leading to aspiration (Murray, 2000). If a patient is unable to produce voicing when they receptively understand the instruction, this may be indicative of possible respiratory problems, vocal fold involvement or laryngeal weakness (Cichero & Murdoch, 2006).

The third item of section 2 evaluates the patient’s ability to perform a volitional dry swallow. A lack of rapid and forceful elevation of the larynx during a dry swallow as well as lack of observed palpitation of the neck during a dry swallow is an indicator of possible increased risk of a swallowing difficulty (Perlman & Schulze-Delrieu, 1998).

### Section 3

The items in section 3 determine the oral motor skills of the stroke participant. The behaviours or functions, as well as structures of the oral mechanism need to be carefully observed during the oral motor exam, as these affect the patient’s ability to chew or swallow safely (Groher, 1997; Shipley & McAfee, 2004). Deficits may affect the oral preparatory phase of swallowing, in terms of poor oral control because of lip and tongue weakness, lingual weakness, reduced range of motion and in-coordination, with bolus formation, manipulation, chewing and swallowing being affected (Langmore, 2001; Shipley & McAfee, 2004). The patient’s ability to cough voluntarily needs to be determined as there is an increased risk for aspiration in patients who have a weakened voluntary cough (Smith-Hammond *et al*., 2001). Observation of facial asymmetry and lip symmetry must be included as facial weakness commonly occurs after a stroke, most typically in the lower facial muscles (Geyer, Gomez, Sheppard & Akhtar, 2009).

### Section 4

This section involves the food trial. The patient must be presented with small amounts (5 ml) of food of different viscosity. Presentation is to progress from consistencies that are the easiest for the stroke patient to manage to the most difficult for the patient to manage (Shipley & McAfee, 2004). Thus, pureed foods (e.g. banana beaten with a fork and mixed with water to form a puree consistency, yogurt and Mageu, a traditional yogurt drink), followed by a soft solid (e.g. mash potatoes, pap, which is similar to mash, and boiled vegetables) and, lastly, a liquid (i.e. water). Pureed foods easily form a bolus and chewing is not required. With a soft solid, chewing is required for bolus formation. Liquids, however, are the most difficult to manage as they do not form a bolus and swallowing requires the least amount of voluntary and reflexive control, increasing the possibility of aspiration (Shipley & McAfee, 2004). Furthermore, unless oral care is good in the patient being assessed, choking on the water will place the patient at risk for aspiration and aspiration pneumonia as a result of bacteria from the oral cavity. Section 4 includes observable signs that can allude to dysphagia when swallowing. These include food spillage, food pocketing, coughing after swallowing and a delayed, absent or painful swallow (Shipley & McAfee, 2004).

### Test administration

The administration of the SADS involves four subsections as noted above. For each item, the administering SLT is required to indicate ‘yes’ or ‘no’ based on the patient’s performance on an item. Each subsection requires either a ‘yes’ or ‘no’ response. Difficulty with any item would result in termination of the screening and the patient is referred for further diagnostic evaluation.

### Pass and fail criteria

The test administrator has the responsibility to pass or fail the patient. Items in section 2 are items that are subjective descriptions and are therefore not referral criteria. A fail on any of the items in section 1, 3 or 4 of the screening tool is indicative of risk of dysphagia, thus a positive result would be indicated on the screening tool. If a fail is indicated in section 3, the screening is to be discontinued. Within section 4, if a fail is indicated in subsection A, the screening is to be discontinued. If a fail is indicated in section 4, subsection B, the screening is to be discontinued. The SADS was designed to be simple and quick to administer. Ease of administration and interpretation were vital prerequisites in the development of the SADS. As part of the study protocol to establish reliability and validity, the results of the SADS were correlated with a subsequent diagnostic dysphagia assessment.

### The pilot study

The pilot study was conducted at a government hospital different to those used in the main study. Upon completion of the screening and diagnostic assessments, the respective SLTs were required to complete a questionnaire probing the content of the SADS. The responses allowed the researcher to determine the need for items to be added, omitted or modified on the screening tool. Based on the results of the pilot study and the questionnaire, the following adjustments were made:

- Modification of the item relating to receptive ability of patient. If the patient presented with poor receptive understanding, the screening was continued and the patient was not referred based on that criterion. This patient was to be assessed with caution.
- Patient position: the patient needs to be positioned with caution, and upright positioning was not a referral criterion.
- Addition of item: volitional swallow.

## Results of main study

There were 63 participants who received the SADS. However, only 62 participants underwent the diagnostic assessment. Hence, the calculations for the screening versus the diagnostic assessment reflect these numbers. All further calculations take this missing frequency into consideration.

From the sample, 30% (*n* = 19) of the participants passed the screening with the SADS, and 69.84% (*n* = 44) were referred from the screening (see Table 2). One stroke participant who was screened was unavailable at the time of the diagnostic assessment and was not followed up. Hence, only 62 stroke participants received the diagnostic dysphagia assessment, results of which are seen in Table 3.

**TABLE 2:** Screening results from the SADS (*N* = 63)

Screening results	*f*	%	Cumulative *f*	Cumulative %
Pass	19	30.16	19	30.16
Fail	44	69.84	63	100

*f*, frequency.

**TABLE 3:** Assessment results from diagnostic dysphagia assessment (*n* = 62).

Assessment results	*f*	%	Cumulative *f*	Cumulative %
Dysphagia	33	53.23	33	53.23
No dysphagia	29	46.77	62	100.00

*f*, frequency.

Results of the diagnostic assessment indicated that 52.23% (*n* = 33) of the participants presented with dysphagia and 46.77% (*n* = 29) of the participants did not present with dysphagia.

### Reliability using Cohen’s kappa

The evaluation of the relationship between the dysphagia screening tool and the diagnostic dysphagia assessment battery was done using correlation coefficients (Schiavetti & Metz, 2002). Cohen’s kappa was used to determine the agreement between two dichotomous variables (Wood, 2007). Using Cohen’s kappa, measures of positive agreement and negative agreement provided information regarding the types of agreement that presented between the screening tool and the diagnostic assessment. The results are presented in Table 4.

**TABLE 4:** Results of Cohen’s kappa

Variable	Pass	Refer	Row total
No dysphagia	18	11	**29**
Dysphagia	1	32	**33**

**Total**	**19**	**43**	**62**

Note: Frequency missing: 1; Percentage of agreement (A): 80.64%; Expected agreements (E): 0.51; Cohen’s kappa: 0.60

The percentage of agreement refers to the agreement between the results of the SADS and the diagnostic assessment (Wood, 2007). Thus, 80.64% of the screenings elicited the correct results (i.e. correct pass and referrals). However, the calculation of percentage of agreement has been critiqued as not being an adequate measure of inter-rater reliability. Thus, Cohen’s kappa, which is a preferred measure of inter-rater reliability, as opposed to percentage agreement, incorporates a calculation of hypothetical probability of chance agreements (Wood, 2007). Thus, the probability that stroke participants presented with dysphagia was calculated. The probability that the participants were referred was also calculated. The calculations of probability were then used to calculate Cohen’s kappa.

The probability that participants were referred based on the screening and presented with dysphagia plus the probability that a patient passed the screening and did not have dysphagia (Wood, 2007) was calculated to be 0.51. Probability for chance agreement refers to the probability that the participant had dysphagia as well as the probability that the participant was referred by the screening and did present with dysphagia was 0.60.

The correlation coefficient for kappa can range from –1.0 to + 1.0; a kappa of 1.0 is indicative of perfect agreement and a kappa of zero shows a poor correlation between the two variables (Wood, 2007). According to Wood (2007), for the purpose of medical studies and diagnosis, a kappa that lies between 0.40 and 0.70 indicates an appropriate inter-rater reliability; the values calculated for this study were thus appropriate, as can be seen in both the percentage of agreement and probability of chance agreement.

## Validity using Cohen’s kappa

Validity is the ability of a test to measure what it is intended to measure (Rust & Golombok, 1999). Content validity (how accurately the questions used in the assessment tool, i.e. the SADS, tap into what is being asked without the response being influenced by other variables), face validity (the degree to which an assessment measures what it appears to measure) and concurrent validity (how well the results of one assessment correlate with another assessment designed to measure the same thing, i.e. the SADS and the diagnostic dysphagia assessment) were calculated (Rust & Golombok, 1999). Concurrent validity relies on timing; hence, the SADS and the diagnostic dysphagia assessment were conducted within 24 hours of each other.

The following measures were used in combination to optimise content validity of the SADS:

- Clinical judgements and input from experienced SLTS working in the area of adult dysphagia were incorporated in the initial conceptualisation of the tool (Schrock & Coscarelli, 2007). These SLTs were not included as part of the sample of SLTs recruited into the study.
- A review of existing dysphagia screening tools in terms of content, as well as each tool’s evidence base, was central to the content of the developed screening tool.
- Each question in the SADS directly assessed a particular objective (i.e. a sign or symptom of dysphagia), allowing direct evaluation of an objective.
- Feedback from the SLT participants confirmed that reading level and use of vocabulary within the SADS were appropriate to enable any health care professional working in an acute health care context to conduct the screening.
- Technical difficulties with instructions and ambiguity of instructions were also considered during the development and construction of the screening tool and were addressed in the pilot study. This did not arise as a concern during the main study.
- Content under-representation, which may interfere with content validity, was avoided by ensuring that the items were adequately able to identify swallowing abnormality and risk of aspiration without indicating severity of dysphagia.

The SLT participants confirmed the overall face validity and appropriateness of the SADS. The percentage of agreement referred to in Table 4 was 80.64%. Thus, concurrent validity of the SADS revealed that it correlated very well with the longer diagnostic assessment conducted. The Cohen’s kappa was used to establish this form of validity.

### Sensitivity and specificity

A measure of sensitivity and specificity of the SADS was calculated. The measures of sensitivity and specificity are binary classification statistical measures. Sensitivity measures the proportion of true positives, which are correctly identified as such (i.e. the percentage of dysphagic patients who are correctly identified as having dysphagia). Specificity measures the proportion of true negatives which are correctly identified (i.e. the percentage of patients with normal swallowing who are correctly identified as not having dysphagia; Haynes, Smith & Hunsley, 2011).

The results were 96.97% sensitivity and 62.07% specificity, as can be seen in Table 5. The results showed the SADS to be sensitive in detecting patients at risk for dysphagia. The specificity of the SADS is lower than its sensitivity; however, the SADS was still deemed to be adequate in identifying patients not at risk for dysphagia, when they do not present with the disorder. The specificity calculation indicates that participants were unnecessarily referred as being at risk for dysphagia when, in fact, they did not present with the disorder.

**TABLE 5:** Sensitivity and specificity of the SADS

Screening result	Assessment result		Total

	No dysphagia	Dysphagia	
Pass	18	1	**19**
	62.07	3.03	
Fail	11	32	**43**
	37.93	96.97	

**Total**	**29**	**33**	**62**
	**46.77**	**53.23**	**100**

*Frequency missing: 1.

### Referral items

There were particular items that were more sensitive in detecting signs and symptoms of dysphagia than others. The items in Table 6 were the most common referral items in the SADS in order of frequency.

**TABLE 6:** Most common referral items and percentage of participants that were referred.

Category	Item and description	%
Section 3: Oral sensory motor examination	4: Facial symmetry	35.7
	3: Lip closure	19.0
Section 1: Observations	1: Level of consciousness	16.6
Section 3: Oral sensory motor examination	5: Tongue range of movement	14.3
Section 4: Food trials	10 & 19: Voice observations after food bolus	4.8 for both
	18: Food spillage	4.8

## Discussion

Analysis of the results suggested that the SADS was a valid screening tool for dysphagia, with high inter-rater reliability. It was specific in being able to detect stroke patients who present with dysphagia. The percentage of agreement score, 80.64%, suggested high concurrent validity, showing that the results from the SADS correlated substantially with the independent diagnostic assessment to which it was theoretically related (Frick, Barry & Kamphaus, 2009). Results that were described showed that minimal and minor modifications were necessary to improve overall content validity of the SADS, which was achieved. Face validity was also acknowledged by the various administrators of the tool, who were qualified clinicians working in adult dysphagia and knowledgeable of contextual challenges and facilitators. A Cohen’s kappa value of 0.6 deemed the SADS as having appropriate inter-rater reliability (0.40–0.70 suggests good inter-rater reliability for medical studies; Wood, 2007).

The sensitivity of the SADS in detecting risk for dysphagia with the chosen population of patients was 96.96%, suggesting high sensitivity (Abramson & Abramson, 2008). Specificity was slightly lower at 62.06%, but, according to Logemann, Veis and Colangelo (1999) that it still fell in the range of 50%–80% meant that it was adequate. The study showed a sensitivity and specificity pattern of high sensitivity and lower specificity (Logemann *et al*., 1999).

There was one false-negative, which was concerning from a dysphagia perspective in terms of complications that could occur from late identification (Heckert *et al*., 2009). It is likely that given the acuteness of the screening (within 24 hours of admission), dysphagia may not have been initially present, as Heckert *et al*. (2009) also found. It is also possible, that further medical deterioration may have occurred subsequent to the screening (Heckert *et al*., 2009). Protocols to prevent such omissions need to be considered. From a time perspective, it was confirmed that the SADS took 10 minutes or less to complete. It was deemed resource conservative and could be easily administered. Whilst the feasibility of the SADS for the current context was not the goal, establishing the validity and reliability empirically was the first step in working toward feasibility. The study was able to establish this.

## Conclusion

The absence of standardised diagnostic dysphagia assessment protocols across government hospitals in South Africa meant that the researchers had to rely on the fact that the protocols used at each hospital in the study were thorough and comprehensive in correctly diagnosing dysphagia in the stroke participants. This was acknowledged as a limitation of the study. An implication of the study is to establish the feasibility of using the SADS in an acute government hospital in South Africa. Whilst different variables were considered in the development of the SADS to facilitate contextual feasibility, this was not an aim of the study. There is therefore a need to establish the exact effects of the context in the implementation of the tool, as the validity, reliability and sensitivity of a measure cannot exist independent of the context.
